# Discernment of possible mechanisms of hepatotoxicity via biological processes over-represented by co-expressed genes

**DOI:** 10.1186/1471-2164-10-272

**Published:** 2009-06-18

**Authors:** Jeff W Chou, Pierre R Bushel

**Affiliations:** 1 Biostatistics Branch, National Institute of Environmental Health Sciences, Research Triangle Park, North Carolina, USA; 2 Department of Biostatistical Sciences, Wake Forest University School of Medicine, Medical Center Blvd., Winston-Salem, NC 27157, USA

## Abstract

**Background:**

Hepatotoxicity is a form of liver injury caused by exposure to stressors. Genomic-based approaches have been used to detect changes in transcription in response to hepatotoxicants. However, there are no straightforward ways of using co-expressed genes anchored to a phenotype or constrained by the experimental design for discerning mechanisms of a biological response.

**Results:**

Through the analysis of a gene expression dataset containing 318 liver samples from rats exposed to hepatotoxicants and leveraging alanine aminotransferase (ALT), a serum enzyme indicative of liver injury as the phenotypic marker, we identified biological processes and molecular pathways that may be associated with mechanisms of hepatotoxicity. Our analysis used an approach called Coherent Co-expression Biclustering (cc-Biclustering) for clustering a subset of genes through a coherent (consistency) measure within each group of samples representing a subset of experimental conditions. Supervised biclustering identified 87 genes co-expressed and correlated with ALT in all the samples exposed to the chemicals. None of the over-represented pathways related to liver injury. However, biclusters with subsets of samples exposed to one of the 7 hepatotoxicants, but not to a non-toxic isomer, contained co-expressed genes that represented pathways related to a stress response. Unsupervised biclustering of the data resulted in 1) four to five times more genes within the bicluster containing all the samples exposed to the chemicals, 2) biclusters with co-expression of genes that discerned 1,4 dichlorobenzene (a non-toxic isomer at low and mid doses) from the other chemicals, pathways and biological processes that underlie liver injury and 3) a bicluster with genes up-regulated in an early response to toxic exposure.

**Conclusion:**

We obtained clusters of co-expressed genes that over-represented biological processes and molecular pathways related to hepatotoxicity in the rat. The mechanisms involved in the response of the liver to the exposure to 1,4-dichlorobenzene suggest non-genotoxicity whereas the exposure to the hepatotoxicants could be DNA damaging leading to overall genomic instability and activation of cell cycle check point signaling. In addition, key pathways and biological processes representative of an inflammatory response, energy production and apoptosis were impacted by the hepatotoxicant exposures that manifested liver injury in the rat.

## Background

The liver is considered one of the vital organs in the body. Its has several major functions including the production of bile to break down fat, glycogen storage, decomposition of red blood cells, production of cholesterol, plasma protein synthesis and drug metabolism just to name a few. The latter takes place by a host of specialized detoxification enzymes and pathways that biochemically modify or metabolize xenobiotics to harmless metabolites and other byproducts for clearance from the body [1]. However, the metabolism of some drugs and compounds leads to toxic intermediates that can harm the liver and severely disrupt its function [2]. Drug-induced liver injury (DILI) is the leading cause of liver failure in the United States (US) and is quickly becoming a major concern world-wide [3]. In fact, DILI accounts for more than 50 percent of the cases of acute liver failure in the US and more than 75 percent of cases of adverse drug reactions result in liver transplantation or death [4]. Drug-induced hepatotoxicity is the most frequent cause for a drug to be withdrawn from the market, restricted in its use or have a warning associated with it due to adverse drug reactions [5]. A better understanding of the pathophysiology of DILI and the mechanisms involved in the manifestation of hepatotoxicity are critical to improving human health and public awareness of potentially harmful toxicants [6].

Microarray gene expression analysis has been used to study the effects of toxicants and other environmental stressors on biological systems [7-11]. Recently, Lobenhofer et al. [12] used gene expression data from rats exposed to a compendium of hepatotoxicants to show that blood gene expression patterns could be used not only to classify animals based on the compound they were exposed to, but also to provide a reasonable indication of the severity level of liver injury. In addition, Bushel et al. [13] demonstrated that gene expression profiles from rat blood samples could accurately predict exposure levels of acetaminophen to the rat liver better than traditional clinical panels. Furthermore, human subjects treated with acetaminophen could be classified based on blood gene expression levels. Although these efforts led to informative conclusions about the genes expression changes related to drug-induced hepatotoxicity, they did not capture the breath of the biological mechanisms altered by the toxic insult.

While hierarchical clustering groups genes and samples by the similarity of expression across all the elements in a data matrix, biclustering [14-17] aims to identify sub-matrices (subsets of rows [genes] and subsets of columns [samples] from an original data matrix) of gene expression that are coherent and possess homogeneity of co-expression. Hence, the biclusters contain co-expressed genes that represent distinct biological responses related to mechanistic changes. In this paper we used a biclustering method to analyze a compendium gene expression dataset containing 318 liver samples from rats exposed to hepatotoxicants (in a dose response and time series manner) and leveraged alanine aminotransferase (ALT), a serum enzyme indicative of liver injury, as the phenotypic marker in order to identify biclusters of co-expressed genes that over-represent biological processes and pathways related to hepatotoxicity.

## Results

### Experimental design

Male 12 week old F344 Fischer rats were exposed to one of the following chemicals: 1,2-dichlorobenzene, 1,4-dichlorobenzene, bromobenzene, monocrotaline, N-nitrosomorpholine, thioacetamide, galactosamine or diquat dibromide [12]. For each chemical, four to six rats (two in one case), were exposed to three or four different dose levels (from subtoxic to toxic) of the compound and sacrificed at 6, 24 and 48 hrs later to extract liver RNA for microarray hybridization. The 318 samples and exposure conditions are listed in Table 1. The number of samples in each group varied from 32 to 72. Liver necrosis was observed in all the rats exposed to one of the eight chemicals at high doses (Table 2). However, 1,4-dichlorobenzene is an isomer of 1,2-dichlorobenzene and is non-toxic at low and mid doses. Since each compound has its unique chemical structure and properties, the activated biological processes, molecular pathways, extent of toxicity in the liver and injury could be highly similar or dissimilar. Considering these variables, we partitioned the columns of the gene expression data matrix into eight groups (one for each chemical) for analysis using Coherent Co-expression (cc)-Biclustering.

**Table 1:** The eight chemical compounds in the compendium

Compound (total number of samples)	Dose (mg/kg body weight)	Time (hour) (number of replicates)		
1,2-dichlorobenzene (34)	15	6 (4)	24 (4)	48 (4)
	150	6 (4)	24 (4)	48 (4)
	1500	6 (4)	24 (4)	48 (2)

1,4-dichlorobenzene (36)	15	6 (4)	24 (4)	48 (4)
	150	6 (4)	24 (4)	48 (4)
	1500	6 (4)	24 (4)	48 (4)

Bromobenzene (36)	25	6 (4)	24 (4)	48 (4)
	75	6 (4)	24 (4)	48 (4)
	250	6 (4)	24 (4)	48 (4)

Diquat (72)	5	6 (6)	24 (6)	48 (6)
	10	6 (6)	24 (6)	48 (6)
	20	6 (6)	24 (6)	48 (6)
	25	6 (6)	24 (6)	48 (6)

Galactosamine (36)	25	6 (4)	24 (4)	48 (4)
	100	6 (4)	24 (4)	48 (4)
	400	6 (4)	24 (4)	48 (4)

Monocrotaline (32)	10	6 (4)	24 (4)	48 (4)
	50	6 (4)	24 (4)	48 (4)
	300	6 (4)	24 (4)	

N-nitrosomorpholine (36)	10	6 (4)	24 (4)	48 (4)
	50	6 (4)	24 (4)	48 (4)
	300	6 (4)	24 (4)	48 (4)

Thioacetamide (36)	15	6 (4)	24 (4)	48 (4)
	50	6 (4)	24 (4)	48 (4)
	150	6 (4)	24 (4)	48 (4)

**Table 2:** Percent of necrosis of the hepatocytes

% of hepatocytes showing necrosis					
Chemical	No Sign	<5%	5%–25%	26%–50%	>50%

1,2-dichlorobenzene	17	8	5	2	2
1,4-dichlorobenzene	31	4	1	0	0
bromobenzene	16	7	5	0	8
diquat	50	10	6	4	2
galactosamine	18	7	8	2	1
monocrotaline	16	11	1	0	4
N-nitrosomorpholine	12	17	2	1	4
thioacetamide	4	18	1	6	7
Total sample size	164	82	29	15	28

### Identification of co-expressed genes based on ALT enzyme levels

Each group of chemicals included a set of samples with different exposure conditions (different doses and time points). We used ALT as a profile to supervise the extraction of the genes that are expressed similarly and correlated with the phenotypic marker. ALT values in each of the chemical exposures were generally elevated from 6, 24, to 48 hrs and from low doses to high doses. For a probability threshold (*p*_*t*_) value of 0.001, supervised cc-Biclustering extracted 84 biclusters in which the genes were correlated (*r*-value >= 0) to ALT and 76 biclusters in which the genes were anti-correlated (*r*-value < 0) to ALT. The number of genes in each bicluster varied. The largest number of genes in a bicluster was 713. The number of groups (chemicals) in the biclusters had a range from a minimum of 1 to a maximum of 8. The biclusters which contained only one chemical could represent the uniqueness in gene expression for the agent and the biclusters which contained all eight chemicals could provide some clues to the common responses to the exposures across the agents.

**Figure 1:**
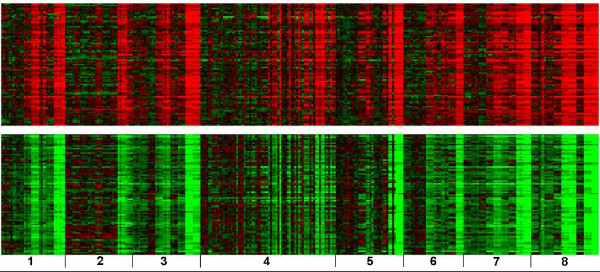
**Heat maps of two biclusters containing samples from all exposures and genes correlated with ALT**. The red color indicates up-regulation and the green color down-regulation. (A) 87 genes up-regulated in the top half and (B) 86 genes down-regulated in the bottom half were significantly correlated and anti-correlated to the ALT profile respectively. From left to right are (1) 1,2-dichlorobenzene, (2) 1,4-dichlorobenzene, (3) bromobenzene, (4) diquat dibromide, (5) galactosamine, (6) monocrotaline, (7) N-nitrosomorpholine and (8) thioacetamide. Each chemical has its dose exposure from low (left) to high (right). Each chemical dose has its time duration from 6 (left), 24 (middle), to 48 hrs (right).

**Figure 2:**
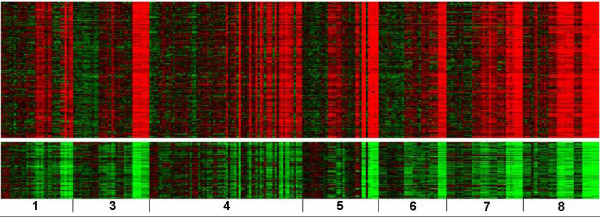
**Heat maps of two biclusters from samples exposed to hepatotoxicants and genes correlated with ALT**. (A) 182 genes up-regulated in the top half and (B) 76 genes down-regulated in the bottom half were significantly correlated and anti-correlated to the ALT profile respectively. From left to right are (1) 1,2-dichlorobenzene, (3) bromobenzene, (4) diquat dibromide, (5) galactosamine, (6) monocrotaline, (7) N-nitrosomorpholine and (8) thioacetamide. Note that 1,4-dichlorobenzene was excluded from these two biclusters. The doses and time points have the same order as in Figure 1.

Figure 1 displays heat maps of two biclusters which included all eight groups of chemicals. There are 87 genes in Figure 1A (the top half) and 86 genes in Figure 1B (the bottom half) significantly correlated and anti-correlated to the ALT profile, respectively. From Gene Ontology analysis of the genes in the biclusters, it was revealed that the significantly over-represented KEGG pathways and biological processes that are up-regulated included general mechanisms such as translation and biosynthetic processes from the genes correlated with ALT and down-regulated categories such as response to external stimuli, negative regulation of coagulation and macromolecule metabolism/biosynthesis from the anti-correlated genes (Table 3). It is interesting that none of the over-represented categories appeared to be related to liver injury. Figure 2 shows heat maps of two biclusters which consisted of seven groups of chemicals not including 1,4-dichlorobenzene. There are 182 genes correlated to the ALT profile in Figure 2A (the top half) and 76 anti-correlated genes in Figure 2B (the bottom half). Gene Ontology analysis revealed that over-represented KEGG pathways and biological processes included a defense response, wound healing, cell proliferation and hematopoietic migration from the genes correlated with ALT and categories related to lipid and steroid metabolism as well as the alkylation of amino acids, tryptophan and sulfur metabolism from the anti-correlated genes (Table 4).

**Table 3:** Significant biological processes and pathways of a general response across all chemicals

**Identifier**	**Term**	**p-value**
**Correlated with ALT**		

GO:0006412	translation	4.45E-04
GO:0044249	cellular biosynthetic process	2.10E-03
GO:0006414	translational elongation	4.31E-03
GO:0006935	chemotaxis	4.99E-03
GO:0022613	ribonucleoprotein complex biogenesis and assembly	4.99E-03
GO:0009058	biosynthetic process	9.04E-03
GO:0009059	macromolecule biosynthetic process	9.27E-03
rno03010	Ribosome	1.55E-06

**Anti-correlated with ALT**		

GO:0006725	aromatic compound metabolic process	1.43E-04
GO:0006519	amino acid and derivative metabolic process	2.13E-04
GO:0050878	regulation of body fluid levels	1.07E-03
GO:0019752	carboxylic acid metabolic process	1.16E-03
GO:0006790	sulfur metabolic process	1.50E-03
GO:0009605	response to external stimulus	2.81E-03
GO:0050819	negative regulation of coagulation	4.87E-03
GO:0006091	generation of precursor metabolites and energy	6.25E-03
GO:0009309	amine biosynthetic process	9.18E-03
rno01040	Polyunsaturated fatty acid biosynthesis	7.97E-03
rno04610	Complement and coagulation cascades	1.27E-02
rno00410	beta-Alanine metabolism	1.41E-02
rno00770	Pantothenate and CoA biosynthesis	7.85E-02

**Table 4:** Significant biological processes and pathways from the hepatotoxicant exposures

**Identifier**	**Term**	**p-value**
**Correlated with ALT**		

GO:0006952	defense response	1.15E-04
GO:0006928	cell motility	8.31E-04
GO:0048519	negative regulation of biological process	1.09E-03
GO:0009605	response to external stimulus	1.17E-03
GO:0018108	peptidyl-tyrosine phosphorylation	1.41E-03
GO:0050819	negative regulation of coagulation	1.46E-03
GO:0018212	peptidyl-tyrosine modification	1.69E-03
GO:0032496	response to lipopolysaccharide	1.80E-03
GO:0007626	locomotory behavior	1.95E-03
GO:0007599	hemostasis	2.01E-03
GO:0008283	cell proliferation	2.05E-03
GO:0009611	response to wounding	2.06E-03
GO:0051707	response to other organism	2.15E-03
GO:0050818	regulation of coagulation	2.19E-03
GO:0002237	response to molecule of bacterial origin	2.19E-03
GO:0051246	regulation of protein metabolic process	3.64E-03
GO:0042127	regulation of cell proliferation	3.91E-03
GO:0065008	regulation of biological quality	5.76E-03
GO:0032501	multicellular organismal process	6.25E-03
GO:0008284	positive regulation of cell proliferation	7.11E-03
GO:0048523	negative regulation of cellular process	7.16E-03
GO:0007596	blood coagulation	8.27E-03
GO:0050789	regulation of biological process	8.48E-03
GO:0050878	regulation of body fluid levels	8.96E-03
GO:0009617	response to bacterium	9.56E-03
GO:0009607	response to biotic stimulus	1.08E-02
rno04640	Hematopoietic cell lineage	3.00E-02
rno04670	Leukocyte transendothelial migration	6.96E-02

**Anti-correlated with ALT**		

GO:0006629	lipid metabolic process	7.39E-04
GO:0032787	monocarboxylic acid metabolic process	5.01E-03
GO:0008202	steroid metabolic process	5.10E-03
GO:0008152	metabolic process	7.68E-03
GO:0042221	response to chemical stimulus	7.86E-03
rno00960	Alkaloid biosynthesis II	2.18E-03
rno00380	Tryptophan metabolism	1.92E-02
rno00920	Sulfur metabolism	4.53E-02

The Gene Ontology analysis of three biclusters from two chemicals, galactosamine and thioacetamide, revealed interesting categorization of the genes (see Additional file 1 Figure S1 and Table S1). The 401 genes in a bicluster subset by galactosamine over-represented a KEGG pathway related to extracellular matrix (ECM) receptor interaction (Figure S1A). Another bicluster of 364 genes subset by thioacetamide had a different set of significantly over-represented categories representing the Wnt signalling pathway and glycerophospholipid metabolism (Figure S1B). The third bicluster of 289 genes subset by both chemicals had biological processes and KEGG pathways related to neuron development and cell adhesion as over-represented categories (Figure S1C).

### Identification of co-expressed genes that discern isomers and an early response

Although the blood serum level of ALT is a good indicator of liver injury, it is not considered a prognosticator of toxic insult as the true nature and extent of the liver damage is not proportional to the elevation in the serum enzyme activity [18]. For instance, a normal ALT level does not necessarily mean that the liver is definitely normal and high levels of ALT in the blood doesn't necessarily indicate the extent to which the liver is inflamed or damaged. In addition, it is likely that ALT levels elevate well after the mechanistic changes have occurred that led to the liver injury from toxic exposure. Therefore, we set out to use cc-Biclustering in an unsupervised fashion (without ALT) so we could find biclusters of genes that may be unrelated to ALT or respond before ALT elevation and yet are very informative in terms of the manifestation of hepatotoxicity. We obtained more biclusters (~ three times more) using unsupervised cc-Biclustering when the same threshold *p*_*t *_was used as in the supervised case.

**Figure 3:**
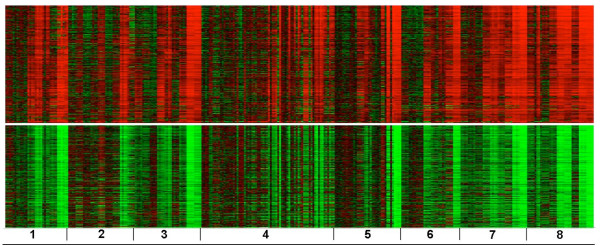
**Heat maps of two biclusters containing all samples and genes unconstrained by ALT**. (A) 330 co-expressed genes are up-regulated in the top half; (B) 409 co-expressed genes are down-regulated in the bottom half. The samples are ordered the same as in Figure 1.

Similar to the biclusters in Figure 1, the genes in the heat maps shown in Figure 3 are subset by all eight chemicals. There are 330 genes up-regulated as shown in Figure 3A (the top half) and 409 down-regulated genes as shown in Figure 3B (the bottom half). Compared to the number of genes in the bicluster shown in Figure 1, the bicluster in Figure 3 contained about four to five times more genes and more significant categories (Table 5). The set of genes that are up-regulated were found to over-represent biological processes and KEGG pathways representative of a more toxic response (i.e. less similar to the general responses over-represented by the genes in the bicluster from the supervised clustering [Figure 1A]). Apoptosis, an inflammatory response and glycolysis/gluconeogenesis were key mechanisms impacted. The set of down-regulated genes contained over-represented categories related to cholesterol biosynthesis, fatty acid metabolism, alkaloid biosynthesis and the peroxisome proliferator-activated receptors (PPARs) signaling pathway. PPAR-α is mainly expressed in the liver and activation of the receptor has been associated with suppression of apoptosis and induction of cell proliferation [19].

**Table 5:** Significant biological processes and pathways of a more toxic response

**Identifier**	**Term**	**p-value**
**Up-regulated**		

GO:0002274	myeloid leukocyte activation	3.04E-05
GO:0002444	myeloid leukocyte mediated immunity	7.08E-05
GO:0045321	leukocyte activation	1.95E-04
GO:0002349	histamine production during acute inflammatory response	1.21E-03
GO:0001821	histamine secretion	1.21E-03
GO:0012502	induction of programmed cell death	1.54E-03
GO:0002532	production of molecular mediator of acute inflammatory response	2.39E-03
GO:0043067	regulation of programmed cell death	2.40E-03
GO:0051052	regulation of DNA metabolic process	2.95E-03
GO:0032496	response to lipopolysaccharide	3.11E-03
GO:0006626	protein targeting to mitochondrion	4.51E-03
GO:0019221	cytokine and chemokine mediated signaling pathway	5.78E-03
GO:0002443	leukocyte mediated immunity	6.19E-03
GO:0006935	chemotaxis	6.58E-03
GO:0006952	defense response	6.91E-03
GO:0043068	positive regulation of programmed cell death	9.83E-03
rno04670	Leukocyte transendothelial migration	2.50E-04
rno00450	Selenoamino acid metabolism	3.71E-02
rno04650	Natural killer cell mediated cytotoxicity	4.56E-02
rno00010	Glycolysis/Gluconeogenesis	7.89E-02

**Down-regulated**		

GO:0006091	generation of precursor metabolites and energy	2.16E-10
GO:0008610	lipid biosynthetic process	2.28E-09
GO:0006695	cholesterol biosynthetic process	6.87E-07
GO:0008203	cholesterol metabolic process	3.62E-06
GO:0006631	fatty acid metabolic process	6.22E-06
GO:0044262	cellular carbohydrate metabolic process	4.21E-04
GO:0042221	response to chemical stimulus	7.85E-04
GO:0005975	carbohydrate metabolic process	8.15E-04
GO:0009896	positive regulation of catabolic process	1.05E-03
GO:0050818	regulation of coagulation	1.40E-03
GO:0007596	blood coagulation	2.68E-03
GO:0045819	positive regulation of glycogen catabolic process	3.03E-03
GO:0007599	hemostasis	3.71E-03
GO:0042493	response to drug	5.78E-03
GO:0002526	acute inflammatory response	8.75E-03
GO:0050819	negative regulation of coagulation	9.20E-03
rno01040	Polyunsaturated fatty acid biosynthesis	2.17E-05
rno04610	Complement and coagulation cascades	3.70E-05
rno00960	Alkaloid biosynthesis II	1.28E-02
rno03320	PPAR signaling pathway	2.31E-02
rno00071	Fatty acid metabolism	2.52E-02

**Figure 4:**
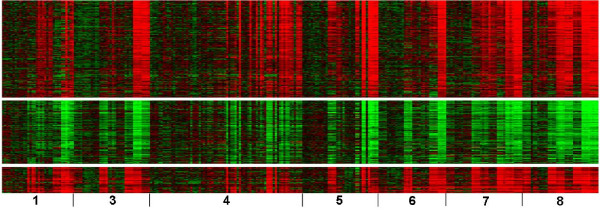
**Heat maps of three biclusters containing genes discerning isomers or depicting an early response to the toxic exposure**. (A) 175 co-expressed genes are up-regulated (top); (B) 114 co-expressed genes are down-regulated (middle). (C) 47 co-expressed were up-regulated (bottom). The samples are ordered the same as in Figure 2.

Heat maps of the gene expression from three biclusters subset by seven chemicals (excluding 1,4-dichlorobenzene), are shown in Figure 4. Figure 4A (the top) shows 175 co-expressed genes up-regulated at a later time and Figure 4B (the middle) 114 co-expressed genes down-regulated. The set of up-regulated genes significantly over-represented categories that are suggestive of liver regeneration (Table 6) [20,21]. Angiogenesis, the regulation of actin cytoskeleton, regulation of adherens (adhesion) junctions and the Toll-like receptor (TLR) signaling pathway were impacted. The down-regulated genes over-represented mechanisms related to energy producing pathways (glucose homeostasis, gluconeogenesis and the pentose phosphate pathways). Comparisons of the over-represented categories between the genes in Figure 3 and 4 clearly differentiate 1,4-dichlorobenzene from the other chemicals since the biclusters in Figure 4 do not contain genes that are co-expressed in the samples exposed to 1,4-dichlorobenzene. Figure 4C (the bottom) had 47 genes up-regulated early and significantly over-represented categories pointing to a negative regulation of protein kinase activity, the mitogen-activated protein kinase (MAPK) signaling pathway, and apoptosis (Table 6).

**Table 6:** Significant biological processes and pathways discerning isomers and from an early toxic response

**Identifier**	**Term**	**p-value**
**Up-regulated**		

GO:0009611	response to wounding	5.10E-05
GO:0001525	angiogenesis	6.07E-04
GO:0002376	immune system process	1.29E-03
GO:0009605	response to external stimulus	1.33E-03
GO:0007596	blood coagulation	3.19E-03
GO:0042730	fibrinolysis	3.56E-03
GO:0006954	inflammatory response	3.96E-03
GO:0030595	leukocyte chemotaxis	4.83E-03
GO:0006935	chemotaxis	4.94E-03
GO:0030195	negative regulation of blood coagulation	6.00E-03
GO:0006873	cellular ion homeostasis	6.72E-03
GO:0042127	regulation of cell proliferation	9.24E-03
rno04660	T cell receptor signaling pathway	2.91E-02
rno04810	Regulation of actin cytoskeleton	3.33E-02
rno04520	Adherens junction	4.58E-02
rno04620	Toll-like receptor signaling pathway	5.95E-02

**Down-regulated**		

GO:0005975	carbohydrate metabolic process	1.89E-04
GO:0032787	monocarboxylic acid metabolic process	1.06E-03
GO:0005978	glycogen biosynthetic process	1.82E-03
GO:0006066	alcohol metabolic process	2.09E-03
GO:0000271	polysaccharide biosynthetic process	5.09E-03
GO:0019318	hexose metabolic process	5.68E-03
GO:0042593	glucose homeostasis	6.71E-03
GO:0019752	carboxylic acid metabolic process	8.06E-03
GO:0006094	gluconeogenesis	8.54E-03
rno04910	Insulin signaling pathway	4.10E-02
rno00030	Pentose phosphate pathway	9.79E-02

**Early response**		

GO:0006469	negative regulation of protein kinase activity	3.82E-06
GO:0016070	RNA metabolic process	3.88E-04
GO:0006355	regulation of transcription, DNA-dependent	4.35E-04
GO:0012501	programmed cell death	6.37E-04
GO:0032774	RNA biosynthetic process	7.12E-04
GO:0031323	regulation of cellular metabolic process	1.27E-03
GO:0045941	positive regulation of transcription	4.03E-03
GO:0030154	cell differentiation	5.41E-03
GO:0006366	transcription from RNA polymerase II promoter	9.35E-03
rno04010	MAPK signaling pathway	2.17E-04
rno04012	ErbB signaling pathway	2.60E-02
rno04912	GnRH signaling pathway	2.67E-02

## Discussion and Conclusion

Over the recent years several ways of investigating drug-induced hepatotoxicity has been explored. Using gene expression analysis of samples exposed to toxicants offers a genome-wide assessment of the transcriptional changes that occur from the insult. However, current methodologies for analyzing the data are limited in that they typically do not "anchor" the changes in gene expression to the phenotype of toxicity nor do they constrain them by the experimental design. Case in point is hierarchical clustering of genes and samples based on gene expression data. Although the method provides an overall view of the clusters of genes that are co-expressed within a group of highly similar samples, it does not extract subclusters of co-expressed genes that are related to a given phenotype, end-point measure of the samples or subset of experiments. Yoon et al. [17] proposed a method for discovering coherent biclusters from gene expression data using decision diagrams constituted from binary representations of a set of samples in which the expression of a subset of genes are highly similar (coherent). However, the method does not integrate phenotypic data nor is it guided by the experimental design (i.e. constraints imposed by a time series or dose response study). Linking co-expressed genes to a phenotype of interest or set of experimental conditions can potentially enhance the interpretation of the biological systems that are impacted from the manifestation of an outcome [22].

We analyzed a compendium gene expression dataset containing 318 liver samples from rats exposed to hepatotoxicants and leveraged alanine aminotransferase (ALT), a serum enzyme indicative of liver injury as the phenotypic marker, to identify several biological processes and molecular pathways that may be associated with mechanisms of hepatotoxicity. Our analysis used an approach to biclustering called Coherent Co-expression Biclustering (cc-Biclustering) for clustering of a subset of genes through a coherent (consistency) measure within each group of samples representing a subset of experimental conditions. Existing biclustering methods use some measure of merit to determine whether a row (gene) or column (sample) should be included or excluded from a bicluster [23]. cc-Biclustering uses a given coherent measure (CM) to determine whether a gene or a group of samples is included or excluded from a bicluster. The CM used between pairs of gene vectors (or gene vectors with a phenotypic profile) is flexible. Depending on a research interest, CM can be chosen to be Pearson correlation, Euclidean distance or some other measure of (dis)similarity. Unsupervised cc-Biclustering uses a pairwise comparison of the gene expression profiles to extract biclusters. In the case of supervised cc-Biclustering, we correlated the co-expression of the subset of genes within a bicluster with ALT. The overlap between the up-regulated genes from the supervised and unsupervised cc-Biclustering methods is 61 genes (Additional file 1; Figure S2) where as the overlap between the down-regulated genes from the two methods is 78 genes (Additional file 1: Figure S3). The sharp contrast between biclusters in Figure 1 and Figure 2 with respect to the over-represented biological categories exhibited by the genes positively correlated with ALT clearly differentiated 1,4-dichlorobenzene from the other seven chemicals. This finding is consistent with a study to predict the levels of necrosis in the rat liver using this same compendium of hepatotoxicants [24]. It was shown that 1,4-dichlorobenzene was the only chemical with an observed necrosis contained in less than 25% of the hepatocytes (one sample was < 25%, 4 samples were < 5%, and 31 samples had no sign of necrosis). All the other chemicals caused observed necrosis in > 50% of the hepatocytes in one or more of the exposed samples (Table 2). Moreover, a bicluster with samples exposed to bromobenzene, diquat dibromide, galactosamine, monocrotaline or N-nitrosomorpholine and 12 genes (including *Cd40, Casp8 *and *Nr4a1*) correlated with ALT and contained over-represented categories related to an inflammatory response, glycolysis/gluconeogenesis and apoptosis (Data not shown). These are biological processes known to be involved in hepatotoxicity [24,25].

Comparison of the over-represented categories between the genes in the biclusters shown in Figures 3 and 4 (both from the unsupervised biclustering) also differentiate 1,4-dichlorobenzene from the other chemicals quite well. In addition, 47 genes in a bicluster were found to be up-regulated early in response to the hepatotoxicant exposure (Figure 4C bottom) and significantly over-represented categories related to negative regulation of protein kinase activity, the MAPK signaling pathway, and apoptosis (Table 6). Many genes in this set (*Atf4, Trib3, Jun, Btg2, Gadd45a, Gadd45b, Ddit3, Cdkn1a, Hmox1, Cdk9, Bag5 and Mcl1*) have been extensively studied in terms of their response to toxicants or other external stimuli [26-31]. Most of these genes are targets of p53 and cause DNA damage. Our finding suggests that the liver injury caused by 1,4-dichlorobenzene could be non-genotoxic while the other toxicants could be genotoxic. This result is consistent with the current finding that early responses of some genes at 6 hr differentiate 1,4-dichlorobenzene from the other seven more toxic chemicals where it is evident that transcriptional regulation by Jun and TP53 leads to necrosis [24].

Although the data from each array is independently collected, in a vast number of cases, many of the related biological samples are highly correlated. For example, some samples are from the same tissue, or treated by the same chemical, or a time series. Same samples are merely biological replicates. In our study we analyzed rat liver samples exposed to one of eight chemicals. Each of the hepatotoxicants has its unique chemical structure and causes different levels and extent of liver injury (Table 2). The activated biological processes and molecular pathways can be highly similar or dissimilar. The gene sets involved in responding to each of the hepatotoxicants can overlap to a large extent or be somewhat unique. Using our biclustering approach we were able to capitalize on the experimental design and/or phenotypic measure to discern possible mechanisms of hepatotoxicity through the over-representation of pathways and biological processes determined by co-expressed genes in cc-Biclusters. More work is underway to efficiently process the wealth of biclusters extracted for a more informed interpretation of the mechanistic changes that take place during the formation of hepatotoxicity.

## Methods

### Gene expression data

Male 12 week old F344 Fischer rats were individually exposed to one of the following chemicals: 1,2-dichlorobenzene, 1,4-dichlorobenzene, bromobenzene, monocrotaline, N-nitrosomorpholine, thioacetamide, galactosamine or diquat dibromide. All eight chemicals were studied using standardized procedures, i.e. a common array platform, experimental procedures and data retrieving and analysis processes. For details of the experimental design see Lobenhofer et al[12]. Briefly, for each chemical, four to six male rats were exposed to a low dose, mid dose(s) or a high dose of the chemical and sacrificed at 6, 24 or 48 hrs later. At necropsy, the liver of the rats were harvested for RNA extraction. A time-matched vehicle control pool was made for each chemical by pooling equal amounts of RNA from each of the control animals. Each treated animal was hybridized against a time-matched control pool to the Agilent Rat Oligonucleotide Microarray with a dye-swap technical replicate. Fluorescence intensities were measured with an Agilent DNA Microarray Scanner and processed with the Agilent Feature Extraction software version A.7.5.1 for bromobenzene and 1,2-dichlorobenzene arrays and version 8.1.1.1 for the others. See the header of the Agilent data files for more details of the scanning and data acquisition hardware and software parameters. The 318 samples and exposure conditions are listed in Table 1. Liver necrosis was observed in all the rats exposed to one of the eight chemicals at high doses. However, 1,4-dichlorobenzene is an isomer of 1,2-dichlorobenzene and is non-toxic at low and mid doses. Since each compound has its unique chemical structure and properties, the activated biological processes, molecular pathways, type of toxicity in the liver and injury could be highly similar or dissimilar. Considering these variables, we partitioned the columns of the gene expression data matrix (Additional file 2) into eight groups (one for each chemical) for analysis using cc-Biclustering (Additional file 3). The number of samples in each group varied from 32 to 72. The data is publicly available at the Gene Expression Omnibus (GEO) database  under series GSE15785 and at the Chemical Effects in Biological Systems (CEBS) database  under accession number 001-00001-0020-000-4.

### Clinical chemistry

At sacrifice, blood was collected into serum separation tubes (BD Microtainer^® ^Tubes, BD, Franklin Lakes, NJ) and serum was separated. Clinical chemistry analyses of alanine aminotransferase (ALT) was performed on all rats at study termination. Serum levels of the established liver injury marker ALT (Additional file 4) increase when the liver shows inflammation, injury or hepatotoxicity.

### cc-Biclustering

Rather than obtaining coherent measures across the whole vector of gene expression, cc-Biclustering uses the sample information in the study design of the experiment to partition the M columns into J groups according to a given phenotypic response or biological study of interest. For instance, as shown in the next section where an expression matrix has eight different chemicals, each group corresponds to a chemical with which samples (biological replicates included) are treated at different doses and time points. A gene expression value of an element in matrix **A **now has four indices, i.e.

(1)

where row index *i *is from 1 to N number of genes; the column index breaks up into three sub-indices, *j*, *k*, and *l*. The index *j *is from 1 to J number of groups (i.e., each of the groups corresponds to a chemical). The index *k *is from 1 to K number of treatments within a group (i.e., corresponding to a given chemical, a treatment is exposure to a given dose at a given time point). The index *l *is from 1 to L, where L is the number of biological replicates. The general idea of cc-Biclustering is to map matrix **A **to a binary coherent matrix **H**(***h*_*i*, *j*_**) according to an inclusion\exclusion criterion function. The **H **matrix has N rows and J columns.

The advantages of bracketing samples or conditions into a set of groups are four-fold. (1) A coherent measure of expression is computed within a group to determine if a gene ***a***_*ij *_is included in a bicluster, where ***a***_*ij *_is a vector of expression values of *i*^*th *^row and *j*^*th *^group. (2) Gene expression ***a***_*ij *_can be time and/or dose series derived. If there were several replicates at a given time and dose, ***a***_*ij *_is evaluated to determine if it is differentially expressed and should be included in a bicluster. (3) The coherent measure (CM) used between pairs of gene vectors ***a***_*i*1,*j *_and ***a***_*i*2,*j *_(or phenotypic profile ***S***_*j*_) is flexible. Depending on a research interest, CM can be chosen to be Pearson correlation, Euclidean distance, or some other measure of biologically relevant similarity. (4) The cc-Biclustering algorithm is simple. Like many coherent value based models (such as an additive model, multiplicative model or based on a given CM) cc-Biclustering converts a gene expression matrix **A **to a binary matrix **H **with a given *p*-value threshold. The extracted biclusters then have constant rows and columns [15]. We used the DAVID database (April 2008 version) for Gene Ontology (January 2008 download) biological processes and KEGG pathway (January 2008 download) analyses of the genes within the biclusters and used p-values for over-representation of these biological categories based on a one-tailed Fisher exact probability or EASE score [32]. The supervised and unsupervised cc-Biclustering algorithms are described in more detail and depicted in pseudo code (Additional file 1). A comparison of the analysis of a microarray gene expression data set from *Arabidopsis thaliana *samples treated with various conditions and times of exposure using unsupervised cc-Biclustering and the Cheng and Church biclustering algorithm [33] with parameters δ = 0.5, α = 1.2 and output = 10 biclusters revealed that our method produced the top 3 biclusters of genes (by size) which are highly correlated within the subset of samples that shared similar exposure conditions with a Pearson correlation in the range of +0.73 to +0.84 whereas the other method produced the top 3 biclusters of genes with correlation in the range of +0.01 to +0.55.

## Authors' contributions

JWC and PRB designed the strategy of cc-Biclustering. JWC implemented the cc-Biclustering algorithm, analyzed the data and wrote part of the manuscript. PRB provided suggestions, advice and guidance for the concept of the research, interpreted the results and also wrote part of the manuscript. The authors have read and approved the final manuscript.

## Supplementary Material

Additional file 1 **Supervised and unsupervised cc-Biclustering details, pseudo code and supplemental figures and table.** Click here for file

Additional file 2 **Two-dimensional matrix of the gene expression ratio data (data preprocessed and fluor-flips per biological sample merged [averaged]).** Click here for file

Additional file 3 **Sample information denoting biological replicates for cc-Biclustering.** Click here for file

Additional file 4 **ALT data for the biological samples.** Click here for file
