# Supplementary material for: Discernment of possible mechanisms of hepatotoxicity via biological processes over-represented by co-expressed genes
Source: BMC Genomics. 2009 Jun 18;10:272. doi: 10.1186/1471-2164-10-272 (PMC2706894; doi:10.1186/1471-2164-10-272)
Supplement: Additional file 1 — Supervised and unsupervised cc-Biclustering details, pseudo code and supplemental figures and table. [file 1471-2164-10-272-S1.pdf]

Additional file 1 – Supplemental materials

Discernment of possible mechanisms of hepatotoxicity via biological processes over-represented by co-expressed genes

Jeff W. Chou[1,*] and Pierre R. Bushel[1,§]

[1]Biostatistics Branch, National Institute of Environmental Health Sciences, Research Triangle Park, North Carolina, USA

[§]Corresponding author
National Institute of Environmental Health Sciences
Biostatistics Branch
P.O. Box 12233
Research Triangle Park, North Carolina 27709
Tel: 919-316-4564
Fax: 919-316-4649

* Current address
Department of Biostatistical Sciences
Wake Forest University School of Medicine
Medical Center Blvd.
Winston-Salem, NC 27157

Email addresses:
JWC: jchou@wfubmc.edu
PRB: bushel@niehs.nih.gov

The gene expression data were first normalized by systematic variation normalization (SVN) using a polynomial fit with 3 degrees [1]. Briefly, the background value for each channel (the low end points determined from the intensity histogram distributions), for each array was determined to be within a range of 20 to 100 in pixel intensity. The first step in SVN is to subtract a background value (approximately 60) from a given array. Before non-linear regression, the intensity measure for each gene and each channel is log base 2 transformed. The log base 2 intensities from the arrays have a mean value. These mean values may vary among the different arrays. Multiarray normalization was performed in order to scale the array data such that their log base 2 intensity mean values are equal to each other. The normalized gene expression intensity values were then converted to ratios, fluor-flips from biological replicates merged (averaged) and then compiled to form a matrix A (Additional file 2). Each expression value has four indices, i.e.

$$a_{i,j,k,l} \qquad (1)$$

where row index i is from 1 to N number of genes and column index breaks up into three sub-index, i.e., subgroup index j is from 1 to J number of conditions; index k is from 1 to K number of treatments within a subgroup j; and replicate index l is from 1 to L for a given index k (Additional file 3). The general idea of cc-Biclustering is to map matrix A to a binary coherent matrix H($h_{i,j}$) according to an inclusion\exclusion criterion function.

Supervised cc-Biclustering algorithm

In the case of supervised cc-Biclustering, index i is from 1 to N and j is from 1 to J, number of subgroups.

$$h_{i,j} = \begin{cases} 1\, if\ CM(a_{i,j}, S_j) > p_t\ and\ r > 0 \\ 0 \qquad\qquad else \end{cases}, \qquad (2)$$

where $\boldsymbol{a_{i,j}}$ is a sub-profile at i^{th} row and j^{th} subgroup which consists of all the expression values of treatment index k from 1 to K and their replicate index l from 1 to L and the S_j is the jth phenotypic anchoring measure S. CM represents a coherent measure between these two sub-profiles. In this paper, CM is a p-value of their Pearson correlation. The correlation r-value set to be larger than 0 for positive correlation only (to get anti-correlated biclusters, one may use a negated anchoring measure S). p_t is a user-defined threshold for the p-value in the range of [0,1]. In such, one gets a coherent measure matrix H($h_{i,j}$) consists of N rows and J columns. To extract supervised coherent biclusters, an exclusive-row approach is used (i.e. genes in a particular bicluster have the same set of subgroups j in which all the $h_{i,j} = 1$). Mapping subgroup index j to the expression data, the corresponding biclusters are obtained which contain subset of genes with the same selected subgroups. As a result, the selected genes with the selected subgroups are highly correlated with the phenotypic measure. The following is the supervised cc-biclustering algorithm:

Supervised cc-Biclustering algorithm

Input: expression matrix A(N,M), supervised profile S, sample information
Columns partition into J subgroups
Set thresholds p_t
Create an empty coherent measure $h_{i,j}$
FOR each profile i
    FOR each subgroup j
        compute the p-value and r-value of correlation measure ($a_{i,j}$, $S_{i,j}$)
            if ($p < p_t$ and $r > 0$) $h_{i,j} = 1$
            else $h_{i,j} = 0$
Create constant biclusters { $h_{i,j} = 1$} and map to gene expression data to get cc-biclusters

Unsupervised cc-Biclustering algorithm

The unsupervised cc-Biclustering approach follows the same idea as the supervised one except that it uses a pair-wised approach instead of anchoring to a phenotypic measure. As such, the binary coherent matrix H has its row indexes i from 1 to N(N+1)/2. The $h_{i,j}$ value is calculated from two different rows $i1$ and $i2$ of expression matrix A(N,M) of jth sub-group. In this unsupervised case, equation (2) is modified to

$$h_{i,j} = \begin{cases} 1\, if\ CM(a_{i1,j}, a_{i2,j}) > p_t\ and\ r > 0 \\ 0 \qquad else \end{cases}, \qquad (3)$$

where $\boldsymbol{a_{i1,j}}$ and $\boldsymbol{a_{i2,j}}$ were two different sub-profiles of expression values at $i1^{th}$ and $i2^{th}$ rows respectively and j^{th} subgroup. To extract constant biclusters from that pair-wised binary coherent matrix H, a gene can be assigned to many of different biclusters. To avoid such, we only assign a gene to a bicluster(s) which has the largest number of subgroups.

In such a bicluster with given set of sub-groups j, a gene at least has another gene with which they are significantly correlated. However, one may also find some genes within this bicluster are not significantly correlated each other. Because of this, we consider these biclusters are “subgroup-selected-biclusters”. The next step in a tandem analysis, one can partition these genes into different clusters using a conventional clustering method. In this study, we used EPIG [2] to these subgroup-selected-biclusters to cc-biclusters in which genes are significantly correlated each other or co-expressed with a given set of subgroups. Briefly, EPIG extracts a set of discrete patterns and then categories each of significant genes to one of the patterns. A gene set of a given pattern

have their expression highly correlated to the pattern profile. The following is the unsupervised biclustering algorithm:

Unsupervised cc-Biclustering algorithm

Input: expression matrix A(N,M), sample information
Columns partition into J subgroups
Set thresholds p_t
Create an empty coherent measure $h_{i,j}$, i = 1 to N(N+1)/2
Set i = 1
FOR each profile *i1*, *i1* = 1 to N-1
    FOR each profile *i2*, *i2* = *i1*+1 to N
        FOR each subgroup *j*
            compute the *p*-value of correlation measure ($a_{i1,j}$, $a_{i2,j}$)
            if ($p < p_t$ and $r > 0$) $h_{i,j} = 1$
            else $h_{i,j} = 0$
            $i = i + 1$
Create constant subgroup-selected-biclusters {$h_{i,j} = 1$} in which genes have largest number of subgroups
Apply EPIG method to subgroup-selected-biclusters for row-wise separation to get cc-biclusters

Table S1. KEGG pathway overrepresented by genes in ccBiclusters

Identifier	Term	p-value
Galactosamine samples bicluster		
rno01430	Cell Communication	2.81E-03
rno00230	Purine metabolism	4.39E-03
rno04512	ECM-receptor interaction	2.34E-02
Thioacetamide samples bicluster		
rno04310	Wnt signaling pathway	6.93E-03
rno00564	Glycerophospholipid metabolism	5.20E-02
rno04530	Tight junction	7.57E-02
rno04740	Olfactory transduction	8.99E-02
Galactosamine & Thioacetamide samples bicluster		
rno04612	Antigen processing and presentation	1.78E-03
rno04540	Gap junction	4.08E-03
rno04514	Cell adhesion molecules (CAMs)	7.49E-03
rno04510	Focal adhesion	2.85E-02
rno04512	ECM-receptor interaction	2.96E-02
rno04940	Type I diabetes mellitus	9.16E-02
rno04360	Axon guidance	9.75E-02

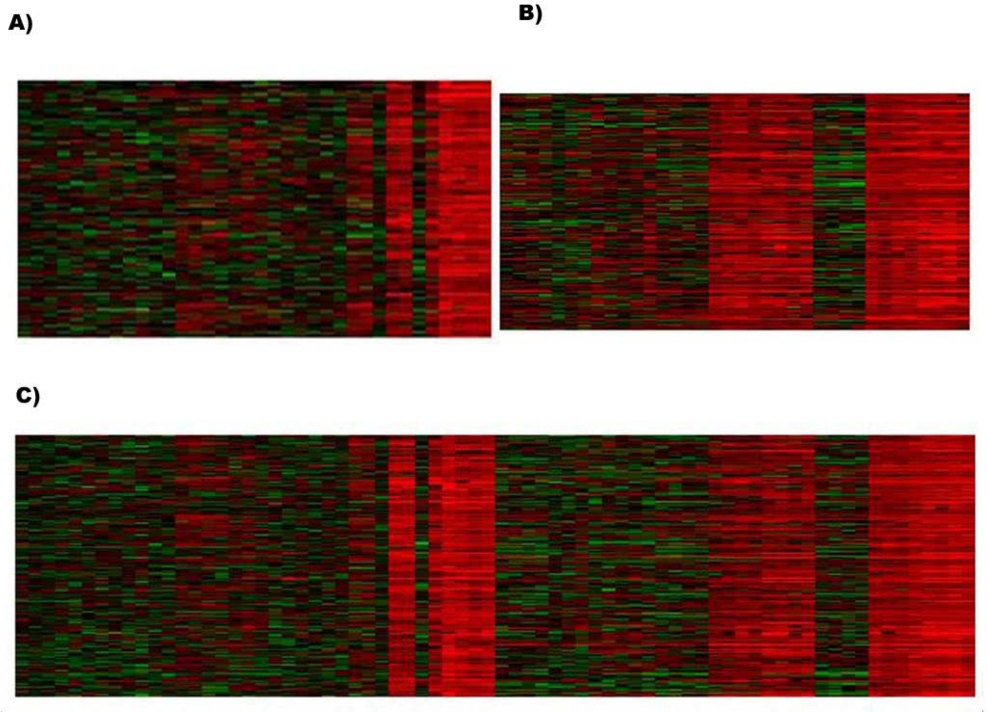

Figure S1. Heat maps of three biclusters containing samples from Galactosamine exposures (A), Thioacetamide (B), either of the two chemicals (C) and genes correlated with ALT. The samples are ordered by chemical exposure as denoted in the legend to Figure 1.

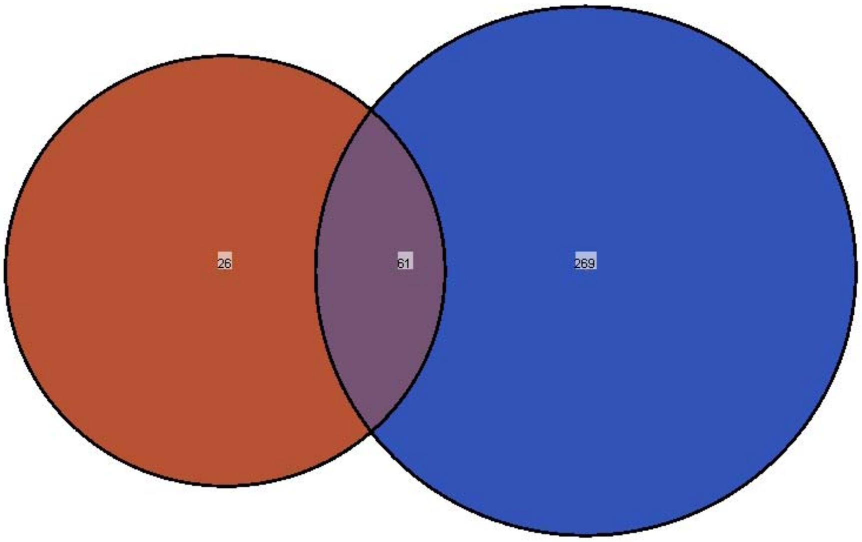

Figure S2. Overlap of up-regulated genes from supervised (left) and unsupervised (right) ccBiclustering.

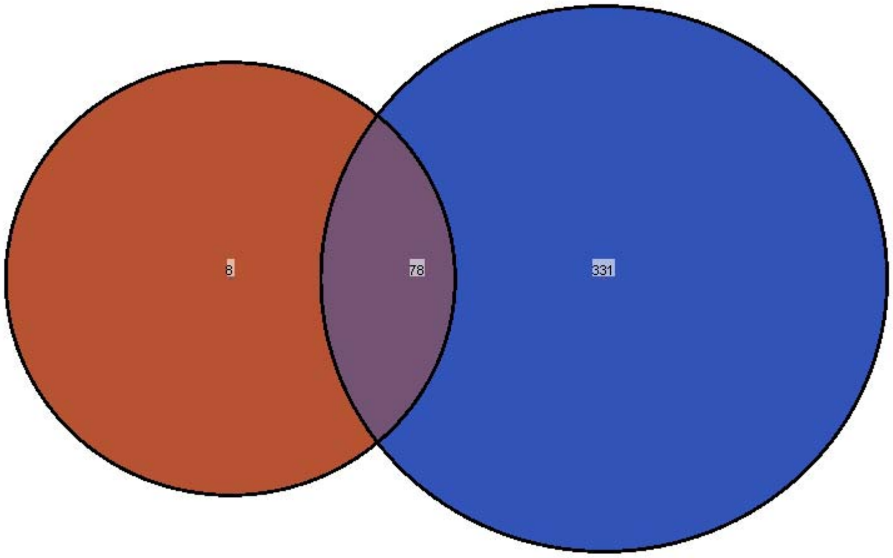

Figure S3. Overlap of down-regulated genes from supervised (left) and unsupervised (right) ccBiclustering.

References

1. Chou JW, Paules RS, Bushel PR: **Systematic variation normalization in microarray data to get gene expression comparison unbiased**. *J Bioinform Comput Biol* 2005, **3**(2):225-241.
2. Chou JW, Zhou T, Kaufmann WK, Paules RS, Bushel PR: **Extracting gene expression patterns and identifying co-expressed genes from microarray data reveals biologically responsive processes**. *BMC Bioinformatics* 2007, **8**:427.
